# Human Cadaveric Retinal Cultures: An Experimental Tool for Retinal Regeneration

**Published:** 2011-01

**Authors:** Kavita G Marita

**Affiliations:** Department of Physiology, GSL Medical College, Lakshmipuram, Rajahmundry, India

**Dear Editor,**

One century ago, Ramon y Cajal^1^ recognized the great value of the retina as a genuine neural center. The retina is a part of the brain itself, evaginating from the lateral wall of the neural tube during embryonic development. As an extension of the central nervous system, the retina, “the miniature brain”, with its multiple neuronal types is considered as a promising experimental model to better understand the function of the brain.^2^ Retinal ganglion cells generate voltage-activated currents in which visual information is coded as they propagate from the retina to higher centers within the brain. The retina, in view of its location, distinctive neuronal morphology, architectural regularity, and accessibility of inputs and outputs, serves as an excellent experimental model.

The retina serves as a suitable model for studying neuronal survival and connectivity in both adults and during developmental stages.^3^,^4^ Being a thin layer, it also well suits experimentation both *in vivo* and *in vitro.* Entire reconstruction of retinal morphology can be performed while preserving its ultrastructure and cell-to-cell relationships. The culture system also provides a useful method to study basic neuronal properties and development of complexity under experimental conditions.^5^,^6^ In recent years, retinal cultures have been routinely used for understanding neural growth, differentiation, gene expression, cytotoxicity, and cell death.^7^,^8^ Retinal cultures are now a widely used tool with broad applications in the field of ophthalmology.

In an experimental study, we used two globes from two male cadavers aged 68 and 84 years to obtain human retinal cultures. The interval between death and enucleation was 2 hours and the interval between enucleation and harvesting cells was 13 hours. Globes were collected from Ramayamma Eye Bank, LV Prasad Eye Institute, Hyderabad, India.

## Retrograde Labeling Of Retinal Ganglion Cells

First, 20 μL of 0.1% fast blue (Sigma-Aldrich, St Louis, MO, USA), a retrograde tracer, was injected in the optic nerve stump. The eyeballs were incubated at 37°C in 5% CO_2_ (Heracell 150 incubator, Thermo Fischer Scientific, Dreieich, Germany) in Hank’s balanced salt solution (HBSS) for 15 hours. The eyeballs were suspended in HBSS leaving the optic nerve stump intact. After 15 hours, the eyeballs were removed from the incubator and allowed to reach room temperature under a laminar flow hood (Class II, Klenzaids, Mumbai, India). The eyeballs were washed with fresh HBSS at room temperature and retinal cultures were prepared.

## Establishing Retinal Cultures

Retinal cultures were prepared according to a previously described method.^9^,^10^ Eyeballs were punctured at the posterior pole adjacent to the optic nerve stump. The eyeballs were split open using a pair of fine forceps to dissect the retina. The retina was then mechanically dissociated using an 18 G needle in D-MEM (Dulbecco’s Modified Essential Medium, Invitrogen, Carlsbad, CA, USA). Retinal cells were seeded on 65 mm culture dishes (Nunc, Langenselbold, Germany) at a density of 1.5×10^5^ cells. Cells were grown in D-MEM supplemented with 10% fetal calf serum (Gibco-BRL, Carlsbad, CA, USA) in an incubator (Heracell 150 incubator, Thermo Fischer Scientific, Dreieich, Germany) with 5% CO_2_ at 37°^C^. The medium was changed on alternate days and cultures were maintained for 30 days.

## Identification of Retinal Ganglion Cells in Vitro

**Figure 1:**
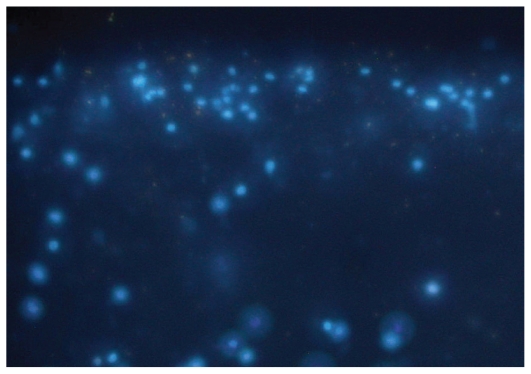
Photomicrograph of fast blue labeled human retinal ganglion cells at 20× magnification.

Retinal ganglion cells (RGCs) were identified on the basis of their soma size.^11^ The soma of RGCs is larger than 12 μM but those of other retinal neurons are smaller than 10 μM (Olympus inverted microscope, CKX 41, DSS Image Tech PVT Ltd., New Delhi, India). We also identified RGCs by traces of retrograde label fast blue under an ultraviolet (UV) filter at excitation of 360 nm and emission of 410 nm (Fig. 1).

**Figure 2:**
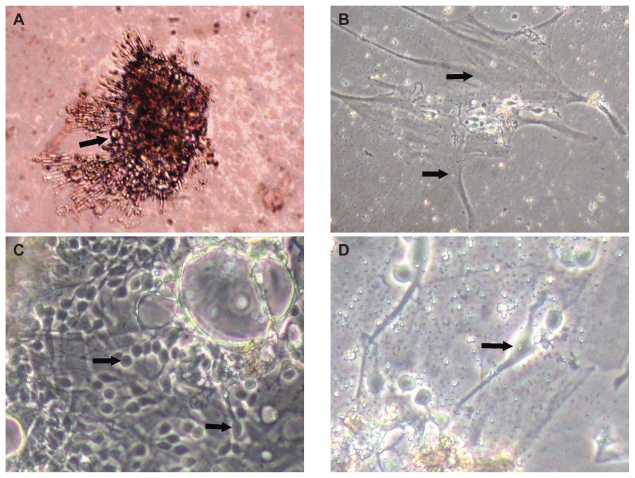
Photomicrographs of cadaveric retinal cultures on various days *in vitro* (DIV). **(A)** at 0 DIV (magnification at 20×); **(B)** at 8 DIV (20×); **(C)** at 16 DIV (40×); and **(D)** at 20 DIV (60×). Arrows show retinal ganglion cells.

Culture characteristics on various days *in vitro* (DIV) included:

- 0 to 4 DIV: flattening of cells from tissue fragments (Fig. 2A)
- 4 to 8 DIV: glial cells (Fig. 2B)
- 8–16 DIV: extension of neurites from clusters and larger neurons, suggestive of ganglion cells (Fig. 2C)
- 20 DIV: individual neurons with extended neurites (Fig. 2D)

The adult human retina contains pluripotent progenitor cells capable of forming neurospheres with different retinal cell types. Kim and Takahashi^12^ were the first to demonstrate a successful explant culture of adult human retina. Explant cultures established from adult human retinae produced 40% healthy and viable cells and were maintained for a period of up to 4 months. Electron microscopic examinations of these cultures revealed that the cells were photoreceptors and neurons with preserved synapses. A resident population of neural progenitor cells in the retina may constitutively replace neurons, photoreceptors and glial cells. Postmortem adult human retinal explants and cell suspensions labeled with 5-bromo-2-deoxyuridine (BrdU), differentiated to express neurofilament M and rhodopsin.^13^

In contrast to the earlier retinal explant cultures by Kim and Takahashi^12^ and postmortem retinal cell suspension by Mayer et al^13^, we established primary retinal cell cultures in uncoated culture dishes. The retinal cells survived approximately 15 hours after death of the individual and enucleation. Retinal cultures prepared after enucleation were maintained for up to 30 DIV without poly-L-lysine or collagen as a matrix. By 8 DIV, the retinal cells started differentiating with flattened glial-like cells on which clusters of neural cells anchored. By 16 DIV, cells formed extensive neural networks, demonstrating synaptogenesis. Traces of the retrograde tracer, fast blue, was incorporated in their soma. Individual viable cells with extended neurites were visible by 20 DIV. Based on soma size larger than 12 μM and extended neurites, the cells were identified as RGCs.

The RGCs established extensive neural networks with significant viable cells, confirming their potential for regeneration postmortem. We have established a simple and novel technique for primary retinal cultures from adult human cadaver eyes. As the retinal cells could survive without matrix in the culture system, this study holds great potential for *in vitro* experiments on RGCs and possible retinal ganglion cell transplants in degenerative ophthalmic conditions with RGC degeneration. Further studies are warranted to specifically characterize the nature and function of cadaveric retinal cells *in vitro*.

## References

[1] Cajal SR (1893). La retine des vertebres. La Cellule.

[2] DeVries SH, Baylor DA (1993). Synaptic circuitry of the retina and olfactory bulb. Cell.

[3] Cajal SR (1893). La retine des vertebres. Trab Lab Invest Biol Univ Madrid.

[4] Ehinger B, Dowling JE, Byorklund A, Hokfelt T, Swanson LW (1987). Retinal micro-circuitry and transmission. Handbook of chemical neuroanatomy Vol 5: Integrated systems of the CNS Part 1.

[5] Akawaga K, Barnstable CJ (1987). Selective localization of glycine-accumulating cells in reaggregate cultures of rat retina. Brain Res.

[6] Govindaiah, Shankaranarayana Rao BS, Ramamohan Y, Singh YK, Dhingra NK, Raju TR (2000). Cytochrome oxidase activity in rat retinal ganglion cells during postnatal development. Brain Res Dev Brain Res.

[7] Masland RH, Raviola E (2000). Confronting complexity: strategies for understanding the microcircuitry of the retina. Annu Rev Neurosci.

[8] Masland RH (2001). The fundamental plan of the retina. Nat Neurosci.

[9] Nichol KA, Everett AW, Schulz M, Bennett MR (1994). Retinal ganglion cell survival in vitro maintained by a chondroitin sulfate proteoglycan from the superior colliculus carrying the HNK-1 epitope. J Neurosci Res.

[10] Govindaiah, Shankaranarayana Rao BS, Raju TR (2002). Enhanced metabolic activity coincides with survival and differentiation of cultured rat retinal ganglion cells exposed to glutamate. Neuroscience.

[11] Guenther E, Schmid S, Grantyn R, Zrenner E (1994). In vitro identification of retinal ganglion cells in culture without the need of dye labeling. J Neurosci Methods.

[12] Kim SU, Takahashi H (1988). Tissue culture study of adult human retina neurons. Invest Ophthalmol Vis Sci.

[13] Mayer EJ, Carter DA, Ren Y, Hughes EH, Rice CM, Halfpenny CA (2005). Neural progenitor cells from postmortem adult human retina. Br J Ophthalmol.

